# LDD: High-Precision Training of Deep Spiking Neural Network Transformers Guided by an Artificial Neural Network

**DOI:** 10.3390/biomimetics9070413

**Published:** 2024-07-06

**Authors:** Yuqian Liu, Chujie Zhao, Yizhou Jiang, Ying Fang, Feng Chen

**Affiliations:** 1 Department of Automation, Tsinghua University, Beijing 100084, China; liuyuqian21@mails.tsinghua.edu.cn (Y.L.); zhaocj22@mails.tsinghua.edu.cn (C.Z.); jiangyz20@mails.tsinghua.edu.cn (Y.J.); 2 LSBDPA Beijing Key Laboratory, Beijing 100084, China; 3 The College of Computer and Cyber Security, Fujian Normal University, Fuzhou 350117, China; 4 The Digital Fujian Internet-of-Thing Laboratory of Environmental Monitoring, Fujian Normal University, Fuzhou 350117, China

**Keywords:** spiking neural networks (SNNs), Transformer, distillation, image classification

## Abstract

The rise of large-scale Transformers has led to challenges regarding computational costs and energy consumption. In this context, spiking neural networks (SNNs) offer potential solutions due to their energy efficiency and processing speed. However, the inaccuracy of surrogate gradients and feature space quantization pose challenges for directly training deep SNN Transformers. To tackle these challenges, we propose a method (called LDD) to align ANN and SNN features across different abstraction levels in a Transformer network. LDD incorporates structured feature knowledge from ANNs to guide SNN training, ensuring the preservation of crucial information and addressing inaccuracies in surrogate gradients through designing layer-wise distillation losses. The proposed approach outperforms existing methods on the CIFAR10 (96.1%), CIFAR100 (82.3%), and ImageNet (80.9%) datasets, and enables training of the deepest SNN Transformer network using ImageNet.

## 1. Introduction

Large-scale Transformers [1,2] have recently achieved significant success in tasks such as natural language processing [3,4] and image classification [5,6]. However, as the size of datesets and network architectures expand, the increasing computational cost and high energy consumption have become key challenges in the training of large artificial neural networks (ANNs) [7]. Spiking neural networks (SNNs) employ sparse spike trains across multiple time steps for data representation and processing. The binary and sparse nature of these spikes gives SNNs an edge in energy efficiency and processing speed, which positions them as a promising solution to the challenges of high energy consumption faced when training large-scale Transformer models.

However, at present, direct training cannot be used to construct very deep SNN Transformers, and the performance of the models obtained is significantly lower compared to ANN models with the same number of parameters. The main challenges in training deep SNN Transformers arise from two aspects:

1. Quantization of the ANN feature space through SNN spike firing: In ANNs, the processing of features is conducted using real values, which allows the model to express delicate hierarchical features. SNNs, however, use discrete spike events to transmit information, which limits the precision of the information.
2. Inaccuracies in surrogate gradients leading to error accumulation across layers: As the activation functions of SNN neurons are discontinuous, surrogate gradients are used to approximate the true gradients during backpropagation. As a result, the behavior of gradients at the spike firing points is inaccurate and, with an increase in network depth, these errors gradually accumulate.

To address the two main challenges in training deep SNNs, we propose the following solutions:

1. Transfer structured feature knowledge from a large pretrained ANN to guide the training of the SNN. Distillation is the most effective method for knowledge transfer and mutual supervision training between models, using the large ANN model as a teacher network to distill the student network, SNN.
2. Transform the training of deep SNNs into training multiple shallow SNNs. Layered loss functions can introduce additional multi-level supervisory signals to correct errors caused by surrogate gradients. During distillation, layer-by-layer distillation losses can be designed, where the teacher network distills the student network in a layer-by-layer manner.

The essence of the proposed layer-by-layer distillation approach is to transfer the feature knowledge of ANNs to the additional temporally enhanced and heterogeneous SNNs. A core challenge in the knowledge transfer process is how to match and align the unique time-step spike sequences of SNNs with the continuous real-valued features of ANNs in both time and spatial dimensions.

For this purpose, we propose a layer-by-layer Dual Distillation approach. SNN neurons’ output features include multi-time-step peaks with temporal dependencies. To transfer ANN knowledge to all temporal dimensions of an SNN, we need to compress the time dimensions while preserving the time-dependent information. We design the Dual SNN, whose output is defined as the dual class token. Each Transformer block learns a temporal layer with an attention structure, using self-attention mechanisms to learn the temporal dependencies of the distillation token at different time steps and making up the Dual SNN. When designing the layered distillation loss function, we perform a transformation between the dual class token and the ANN class token to control the intensity of feature aggregation, achieving alignment in the spatial dimension.

Existing work using ANN distillation for directly training SNNs has focused on the ResNet framework, improving model classification accuracy. However, these approaches adopt an end-to-end distillation strategy, aligning the ANN and SNN using only the output features of the final layer, which results in weak supervisory signals and ignores the multi-time dimensional characteristics of SNNs. Additionally, for SNN Transformer networks, the structural consistency with ANNs is disrupted, making it impossible for end-to-end distillation to achieve higher similarity in model features. In contrast, our proposed LDD method, for the first time, addresses the structural inconsistency between ANN and SNN Transformer architectures. By employing layerwise distillation, it ensures that each layer of the SNN is optimally aligned with its corresponding layer in the ANN. The introduction of temporal layers and the Dual SNN allows the transfer of ANN feature knowledge into the extended temporal domain of the SNN. This fine-grained distillation process helps preserve the complex feature details from the ANN.

In summary, our main contributions are as follows:

- We propose layer-by-layer Dual Distillation (LDD) which, for the first time, achieves precise alignment of ANN and SNN features at different levels of abstraction on a Transformer network, mitigating the difficulty in directly training deep SNN Transformer networks.
- We propose the temporal Dual SNN, which imports the feature knowledge of neural networks into the extended time domain and heterogeneous spatial domain of the SNN, solving the difficulties in layer-by-layer distillation caused by multiple time steps and network heterogeneity.
- Compared to other methods for training SNNs, we achieve state-of-the-art performance on standard datasets and train the deepest SNN Transformer network using the ImageNet dateset, achieving efficient sample utilization.

## 2. Related Work

### 2.1. Training of SNNs

ANN training has achieved significant success using gradient-based algorithms [8,9], calculating the gradient of each neuron operator during forward propagation, with errors being backpropagated from the output layer to the input layer. However, SNNs utilize step functions to send neuron outputs into spikes, as seen in integrate-and-fire (IF) and leaky-integrate-and-fire (LIF) neurons [10]. These spikes, represented by binary values over multiple time steps, encode information at different firing times. However, this spiking computation is non-differentiable, necessitating the use of surrogate gradient functions as approximations for the actual gradients, thus allowing SNNs to also be directly trained using backpropagation algorithms such as Spatio-Temporal-Backpropagation (STBP) [11,12]. Nevertheless, surrogate gradients increase the computational load due to their complex calculations and dependencies across multiple time steps, thereby incurring significant computational costs [13]. Additionally, surrogate gradients do not precisely describe the behavior of spiking neurons and the accumulated error in gradients prevents the direct training of deep SNNs.

### 2.2. Transformer and Spike-Based Transformer

The Transformer has shown exceptional performance in tasks such as machine translation [14], text generation [15,16], and speech recognition [17,18]. Its core is the self-attention mechanism [19], which allows the model to allocate weights to different positions in the input sequence, thus better understanding the context of the input sequence. Unlike Long Short-Term Memory (LSTM) networks [20] or Gated Recurrent Units (GRUs) [21], the Transformer completely abandons the recurrent structure, significantly improving computational efficiency and suitability for parallel computation. Compared to traditional convolutional neural networks, the Transformer has a smaller inductive bias, offering greater flexibility and inductive capability.

Spike-based Transformers [22,23], which have been recently designed for direct SNN training, modify two key components of the Transformer: the Vanilla Self-Attention (VSA) and the Multi-Layer Perceptron (MLP). The surrogate gradient employed exhibits inaccuracies in its behavior at the spiking points, leading to a gradual reduction in the precision of the feature space across layers. When SNNs utilize deep Transformer structures to process datesets, their performance is somewhat diminished compared to ANNs. In the absence of external data, the ImageNet dateset only supports the training of spike Transformer networks with no more than ten blocks, resulting in low sample efficiency.

### 2.3. SNN Training with Knowledge Distillation

Knowledge distillation [24,25] aims to transfer the knowledge from a large, complex model (commonly referred to as the “teacher model”) to a smaller, more deployable model (referred to as the “student model”), improving the efficiency of the model and reducing the demand for computational resources while maintaining high performance. Previously, such approaches were mainly applied for the direct training of ANNs, using the teacher model’s output to customize the supervisory signal, providing more detailed guidance compared to hard labels [26]. Distillation operations are generally easier between networks with similar structures [27], as the consistency of the architecture allows the student model to more effectively learn the feature representation and decision boundaries of the teacher model.

Previous research has shown that knowledge distillation can be used to transfer knowledge from large ANNs to SNNs [28,29], and this has been applied in both ANN-to-SNN conversion and direct SNN training efforts. For ANN-to-SNN conversion, setting and adjusting the spiking threshold for each layer is a significant challenge. Through employing distillation, it is possible to fine-tune ANN-to-SNN models, enhancing the accuracy of the transformed models [30,31]. During the threshold balancing process in ANN-to-SNN conversion, soft labels introduced through the trained large ANN model are used to update the threshold of each layer, ensuring that the converted SNN can activate neurons appropriately [32].

For directly training SNNs, distillation is primarily used for residual structures [28,31]. Deep Spiking ResNet and ANN ResNet have similar structures, inherently satisfying the requirements for knowledge transfer through distillation in isomorphic networks. These methods often focus on the output layer for distillation, potentially missing out on valuable intermediate representations learned by the teacher model [33,34]. However, without matching intermediate representations or feature maps between the teacher and student networks, it is unable to handle the temporal dynamic adaptability required for the structural differences between ANNs and SNNs. Going further, for SNN Transformer networks, the structural consistency with ANNs is disrupted, making it more challenging to ensure stability in knowledge transfer within deep networks [35]. To date, there have been no specialized studies on guiding SNNs using the Transformer structure through ANN-based instructions.

## 3. Preliminaries and Problem Analysis

### 3.1. Spike-Based Transformer Model

We use a spike-based Transformer [22], which incorporates the Transformer into the spike-driven paradigm with only sparse addition. It includes four parts: Spiking Patch Splitting (SPS), Spike-Driven Self-Attention (SDSA), MLP, and a linear classification head. The MLP and the linear classification head are identical to those in ANNs.

For the SPS part, input an image $I\in R^{T\times C\times H\times W}$, where T, C, H and W denote the time step, channel, height, and width of the 2D image. First, the Patch Splitting Module (PSM), which consists of multiple convolutional layers, processes and splits the input into N long sequences with D-dimensional channels.

(1) $$\mathrm{PSM}\left(I\right)=\mathrm{Conv}2\;d\left(I\right),\;\mathrm{PSM}\in R^{T\times N\times D}.$$

Another Conv layer is then used to generate Relative Position Embedding (RPE):

(2) $$\mathrm{RPE}=\mathrm{BN}\left(\mathrm{Conv}2\;d\left(\mathrm{SN}\left(u\right)\right)\right),\;\mathrm{RPE}\in R^{T\times N\times D},$$

where SN(u) denotes the layer of spiking neurons that performs spike processing. We combine the outputs of RPE and PSM to obtain the SPS results.

(3) $$U_{0}=\mathrm{PSM}+\mathrm{RPE},\;U_{0}\in R^{T\times N\times D}$$

Then, we pass $U_{0}$ to several blocks of Spike-driven Transformer encoders, whose core is SDSA, utilizing the *Q*, *K*, and *V* spikes to model the global cross-correlation information of the image. After extracting the feature matrices *Q*, *K*, and *V* from the output using linear convolutional layers, the essence of SDSA is to perform masked operations on *Q*, *K*, and *V*.

(4) $$\mathrm{SDSA}\left(Q,K,V\right)=Q⊗\mathrm{SN}\left(\mathrm{SUM}\left(K⊗V\right)\right),$$

where ⊗ is the Hadamard product.

### 3.2. Training Difficulties

To train the Spike-driven Transformer, on the one hand, compared to residual networks, there is a stronger dependency on large datesets, as it is constrained by the relatively small inductive bias of the Transformer structure. In the absence of external data, the ImageNet dateset only supports the training of the Spike-driven Transformer with no more than ten blocks, resulting in low sample efficiency. On the other hand, due to the inaccuracy of the surrogate gradient used in the backpropagation algorithm of SNNs at the points of spike firing, there is a layer-by-layer reduction in the precision of the feature space. When SNNs employ deep Transformer structures to process datesets, their performance suffers in comparison to ANNs. Utilizing the knowledge of the feature space from artificial neural networks to guide each layer helps mitigate the error accumulation caused by inaccurate gradients at each layer.

Self-attention in the Spike-driven Transformer is specially designed for modeling spike sequences. The Q, K, and V are generated through the spike neural layer, which degrades the matrix dot-product calculation to the logical AND operation and summation operation. Therefore, the parameterized layers are different between the ANN and Spike-driven Transformer. Furthermore, the temporal and spatial properties of spiking neural networks require an additional time dimension in the input, resulting in structural differences. It is difficult to achieve ideal results simply by minimizing the feature distributions between the ANN and SNN, due to the lack of sufficient distillation information. Therefore, SNN layer-by-layer distillation through the ANN requires overcoming both the model structure and feature dimension issues.

## 4. Layer-by-Layer Dual Distillation

**Figure 1:**
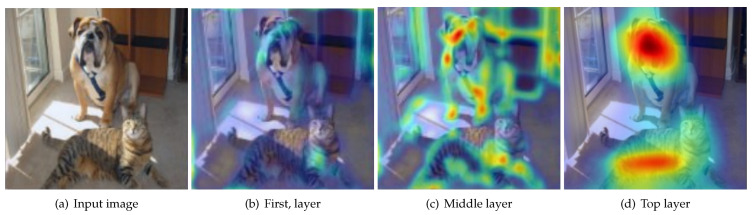
The attention map of different layers in a convolutional neural network.

Previous works have shown that different layers of neural networks capture different attentional information [36]. As shown in Figure 1, the neurons in lower layers are heavily activated by input information and extract simple features, such as edges and colors. As the network progresses to deeper layers, the middle layers begin to extract more complex features, including corner textures. In the top layers, the features extracted are more abstract and typically involve semantic representations of the image. Indeed, the features in the output layer do not fully reflect the complexities of the entire network.

**Figure 2:**
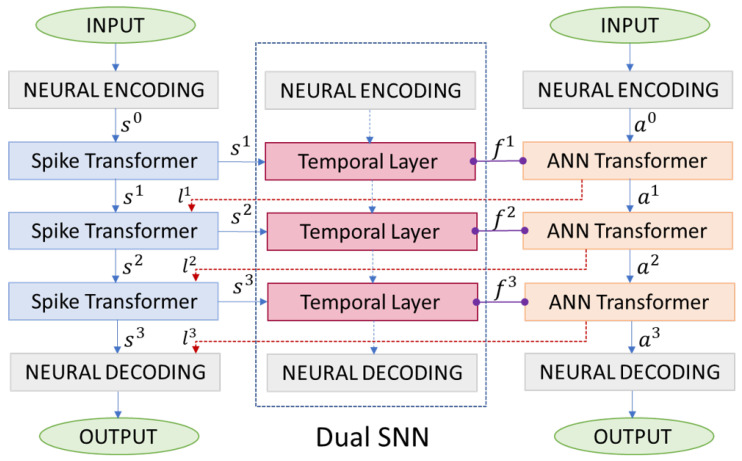
The training pipeline of layer-by-layer ANN-to-SNN knowledge distillation. We propose the Dual SNN (dashed blue box) to project the multiple time dimensions of the SNN into the single time dimension of the ANN. Each Transformer block learns a temporal layer with an attention structure to capture the temporal dependencies of the distilled labels, aligning its output with the ANN features through distillation.

Due to the complex and significantly divergent self-attention mechanisms, the SNN Transformer differs greatly from ANN networks. There is no specialized research on the distillation from ANNs to SNNs within Transformer architectures. Traditional distillation strategies used in the ResNet framework that minimize the differences in output distributions between ANNs and SNNs are not very effective due to the lack of sufficient distillation information. We propose a layer-by-layer supervision strategy, combined with the loss alignment strategy, which provides a robust framework for transferring complex feature knowledge from ANN to SNN while preserving the multi-time dimensional characteristics of SNNs. As input samples pass through each Transformer block, each layer can output feature maps that contain attention information at different levels. A hierarchical alignment distillation strategy is proposed, which extracts attention information layer-by-layer from the ANN. The overall process of layer-by-layer knowledge distillation is illustrated in Figure 2. In this section, we give details about the distillation paradigm with a layer-by-layer supervision strategy and a loss alignment strategy.

### 4.1. Layer-by-Layer Supervision Strategy

The key aspect of the LDD method is to project the SNN into the ANN space along the time dimension. We achieve this through designing the Dual SNN utilizing attention mechanisms. In the SNN Transformer network consisting of *N* blocks, each block corresponds to a temporal layer learning the temporal attention map of the output features, modeling long-term dependencies in the time dimension. The *N* temporal layers together form the Dual SNN, serving as the temporal mapping function of the current SNN into the ANN space.

Each temporal layer has two sub-layers, including a multi-head self-attention layer and a fully connected layer. To project the output features of each block along the temporal dimension, it is necessary to learn a set of vectors, which contain the dependency relationships of the features at different time steps, through the temporal layer. To this end, we treat each temporal dimension of data as input for the multi-head self-attention layer, so that the output vectors will encompass temporal dependencies in all time steps. Subsequently, the projection along the temporal dimension can be achieved through a fully connected layer. Concretely, the pipeline of the *l*-th temporal layer is given by Definition 1.

**Definition** **1** (Temporal Layer)**.** *Let the output features of the l-th SNN block be $S^{l}=\left[s_{1}^{l},\ldots ,s_{N+1}^{l}\right]$$\in R^{(N+1)\times T\times C}$, where $s_{N+1}^{l}\in R^{T\times C}$ is the distillation token. Then, the output of the l-th temporal layer with input $s_{N+1}^{l}$ is given by the following formula:*

(5) $$\begin{array}{cc} {\tilde{h}}^{l} & =\mathrm{SG}(s_{N+1}^{l}),\\ Q^{l},K^{l},V^{l} & ={\tilde{h}}^{l}W_{Q}^{⊤},{\tilde{h}}^{l}W_{K}^{⊤},{\tilde{h}}^{l}W_{V}^{⊤},\\ \mathrm{Temporal}\;\mathrm{Layer}\left(s_{N+1}^{l}\right) & =Linear(Attention\left(Q^{l},K^{l},V^{l}\right)), \end{array}$$

*where the function SG(·) stands for the stop-gradient operation; Attention(·,·,·) stands for the self-attention layer given by*

(6) $$Attention\left(Q,K,V\right)==\mathrm{Softmax}\left(\frac{Q_{F}K_{F}^{T}}{\sqrt{d}}\right)V_{F},$$

*where d=C/H, H is the head number, and Linear(·) stands for the fully connected layer given by*

(7) $$Linear\left(x\right)=xW^{⊤}+b.$$

*The trainable parameters of the temporal layer are $W_{Q}$, $W_{K}$, $W_{T}$, W, and b.*

**Figure 3:**
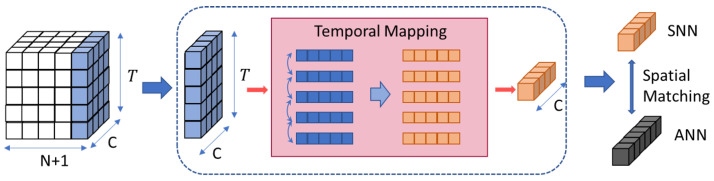
Illustration of the process of aligning features using the temporal layer with a segment length 5 × 5. In the temporal layer, a segment length of 5 × 5 is used to process the temporal attention information in the feature map, projecting it into the single time dimension feature space of the ANN. This produces the Dual SNN token, which is then spatially matched with the ANN class token to compute the distillation loss.

The process of aligning features using the temporal layer is illustrated in Figure 3.

All the temporal layers form the Dual SNN, as shown in Figure 2. The Dual SNN essentially serves as a continuous-valued network projecting multiple time dimensions of SNN into one dimension of ANN. The LDD method incorporates dynamic adjustments during the distillation process, which allows for real-time optimization of the knowledge transfer. This adaptive approach ensures that the discrepancies between the ANN and SNN architectures are minimized throughout the training. By focusing on layer-by-layer distillation, the LDD method precisely align the features of ANN and SNN at different levels of abstraction, which is specifically designed to address the structural inconsistencies between ANNs and SNNs, particularly in Transformer architectures. This results in improved stability and performance of the SNN, which is a significant advancement over previous methods that struggled with such architectural differences.

### 4.2. Loss Alignment Strategy

After connecting to the temporal layers, the alignment loss is introduced to build dependencies of models in the following form:

(8) $$L_{\mathrm{Align}\;}^{\left(k\right)}\left(t,s\right)={∥F_{t}^{\left(k\right)}-F_{s}^{\prime (k)}∥}_{2},$$

where *t* and *s* represent the teacher network ANN and the student network Dual SNN, respectively, and $F_{t}^{\left(k\right)}$ and $F_{s}^{\prime (k)}$ are the feature representations from the k-th blocks of the teacher ANN and the student Dual SNN. Minimizing $L_{\mathrm{Align}\;}^{\left(k\right)}\left(t,s\right)$ enforces the k-th intermediate block of the student model to be similar to the k-th intermediate block of the teacher model. This transfers knowledge from the teacher network to the student network. The total loss function of the LDD framework can be defined as follows:

(9) $$L_{\mathrm{total}\;}=\lambda L_{\mathrm{model}\;}+\left(1-\lambda \right)\sum_{k=1}^{n}L_{\mathrm{Align}\;}^{\left(k\right)}\left(t,s\right),$$

where $L_{\mathrm{Align}\;}^{\left(k\right)}$ is the alignment loss, *n* is the total number of alignment layers, $L_{\mathrm{model}\;}$ is the model loss term, and λ represents the proportion of the distillation component in the original training.

The entire LDD training process is illustrated in Algorithm 1.
**Algorithm 1** Overall training algorithm1:**Input:** Pretrained ANN (teacher model, Ta), SNN (student model, St), input (*X*), target (*Y*), weights (*W*), threshold voltage (*V*), layers (*L*)2:# Forward propagation3:**for** t=1 to *T* **do**4:   $O_{0}^{t}\leftarrow$ Poisson Encoder (X)5:   **for** l=1 to L−1 **do**6:     **if** t==1 **then**7:        $F_{l-1}^{t}=X\left(t\right)$8:     **end if**9:     $F_{s}^{\prime (l)}=\mathrm{Temporal}\;\mathrm{Layer}\left(F_{l}^{t}\right)$10:  **end for**11:**end for**12:# calculate total loss13:$L_{\mathrm{total}\;}=\lambda L_{\mathrm{model}\;}+\left(1-\lambda \right)\sum_{l=1}^{n}L_{\mathrm{Align}\;}^{\left(l\right)}\left(t,s\right)$14:# Backward Propagation15:**for** t=1 to *T* **do**16:   **for** l=1 to L−1 **do**17:     $\frac{dL_{\mathrm{total}\;}}{dU_{l}^{t}}=\frac{dL_{\mathrm{total}\;}}{dF_{l}^{t}}\frac{dF_{l}^{l}}{dU_{l}^{t}}$18:   **end for**19:**end for**

## 5. Approximation Error

The approximation error of the distillation involves two principal aspects: the student network’s fit to the data and the student network’s accuracy in mimicking the behavior of the teacher model. The former error measures the student model’s performance on the training data, namely the discrepancy between its predictions and the actual labels. The latter error assesses the student model’s precision in emulating the teacher model’s behavior. It measures the similarity between the outputs of the student model and those of the teacher model. In our proposed layer-wise distillation approach, the emulation of the teacher model’s behavior is accomplished by the Dual SNN.

To analyze the approximation errors in layer-wise and end-to-end distillation, consider the impact of each neuron on the overall network error and analyze how the approximation error of a certain layer propagates through the remaining hidden layers, as presented in Theorem 1.

**Theorem** **1.** *Consider an L-layered SNN trained by end-to-end distillation. $J_{l}$ is the Jacobian matrix of layer l, expressing the dependency of each layer’s neurons on the previous layer. The approximation error ε satisfies*

(10) $$\varepsilon =\sum_{i,j}\frac{1}{c}\sum {\left(\left|V_{0}\right|\sum_{l=L}^{i+1}E\left[\left|J_{l}\right|\right]\right)}_{*,j}\left(E_{\left[\left|e_{i,j}\right|\right]}+E\left[\left|\epsilon_{i,j}\right|\right]\right),$$

*where $e_{i,j}$ represents the classification error of a neuron (i,j), $\epsilon_{i,j}$ represents the distillation error of the neuron, and $V_{0}$ is the weight matrix of the output layer.*

As for SNN, similarly to [37], accumulating the approximation errors of all neurons can yield the fitting error of the directly trained network with respect to the data. This portion of the error is related to the number of network layers *L*, the number of samples *N*, and the Lipschitz constant of the network function $L_{f}$.

**Proposition** **1.** *Given an SNN with L hidden layers, the expected generalization error of the network can be upper bounded as follows:*

(11) $$\sum_{i,j}\left(E_{\left[\left|e_{i,j}\right|\right]}\right)\leq \exp\left(-\frac{L}{2}\log\frac{1}{\eta }\right)\sqrt{\frac{L_{f}{\left|y\right|}_{\max}}{N^{2}}},$$

*where L is the number of layers in SNN, $\eta ={\left(E_{w_{1},\ldots ,w_{L}}\left(\prod_{k=1}^{L}\eta_{k}\right)\right)}^{\frac{1}{L}}<1$ is a constant depending on the average information loss of each layer, $L_{f}$ is the Lipschitz constant of f on D, and N is the number of samples.*

Although layer-wise distillation does not change the network structure and does not affect the original function-fitting ability of the network, it shortens the propagation path of distillation errors in each layer. The distillation error of the network is then transformed into the product of mutual information entropy at each layer and the neuron distillation approximation error. The overall approximation error of the network is represented in Theorem 2.

**Theorem** **2.** *Given an L-layered SNN which is trained by LDD, the approximation error consists of two parts: fitting the real data distribution with a finite sample and fitting the teacher network distribution. It satisfies the following:*

(12) $$\varepsilon =\sum_{i,j}\frac{1}{c}\left(\lambda \sum {\left(\left|V_{0}\right|\sum_{l=L}^{i+1}E\left[\left|J_{l}\right|\right]\right)}_{*,j}E_{\left[\left|e_{i,j}\right|\right]}+\left(1-\lambda \right)\sum E{\left[\left|Q_{l}\right|\right]}_{*,j}E\left[\left|\epsilon_{i,j}\right|\right]\right),$$

*where $Q_{l}$ is the mutual information entropy of each layer between the Dual SNN and the teacher network, $J_{l}$ represents the dependency of each layer’s neurons on the previous layer, $e_{i,j}$ represents the classification error of a neuron (i,j), and λ represents the proportion of the distillation component in the original training.*

Thus, for a learning problem, the argument centers on whether the following inequality holds:

(13) $$\sum E{\left[\left|Q_{l}\right|\right]}_{*,j}\leq \left|V_{0}\right|\sum_{l=L}^{i+1}E\left[\left|J_{l}\right|\right].$$

The validity of this inequality indicates that the LDD method, compared to end-to-end distillation, reduces the dependency of errors between successive layers, thereby decreasing error accumulation and lowering the overall approximation error of the network. This inequality demonstrates the advantages of LDD:

1. Fine-grained knowledge transfer—layer-wise distillation involves transferring knowledge between the corresponding layers of the teacher and student models, enabling the learning of representations in intermediate layers.
2. Traditional deep SNN training faces the issues of gradient vanishing or explosion, especially in very deep networks. Through employing layer-wise distillation, the training of a deep network can be decomposed into multiple shallow training stages, each corresponding to a part of the network. This effectively alleviates the accumulation of gradient errors caused by surrogate gradients and dependencies over multiple time steps.

## 6. Experiments

In this section, we evaluate the proposed LDD method on three benchmark datesets, CIFAR10, CIFAR100, and ImageNet. To further show the advantage of training the deep SNN Transformer, LDD was employed to train the currently deepest SNN Transformer network. Moreover, we conducted ablation experiments to validate the effectiveness of the temporal layers and the Dual SNN.

The student SNN is trained from scratch, and we use the Vit-L/32 as the teacher ANN, which is mentioned in [38]. We use the Adam optimizer in the process of training the student SNN with the layer-by-layer distillation (proposed work). We set the batch size as 256. The training epoch was set as 200, the learning rate was 0.05, the momentum was 0.9, and the weight decay was 0.0001. The decayed term α for attention losses is set to 0.9.

### 6.1. Performance Comparison with Other Methods

Our method can be applied to training SNN with all types of Transformer computations. In practice, we combine LDD with prior SNN Transformer computations. Conducting our method results in a significant improvement in accuracy compared to the original training paradigm. We evaluate the performance of the LDD method on three benchmark datesets, namely CIFAR10, CIFAR100, and ImageNet, and compare with the baseline model trained directly without using the LDD method.

ImageNet [39]: ImageNet, introduced by Jia Deng et al. in [39], is one of the most commonly used datasets for image classification, detection, and localization in the field of deep learning. It contains 1000 classes, with approximately 1000 images per class. The total number of training images is around 1.2 million, with an additional 50,000 images for the validation set and 100,000 images for the test set. The images are 224 × 224 RGB images. The batch size is 128, and the optimizer is AdamW with 350 training epochs. The SPS module divides the image into 14 × 14 patches. Table 1 shows that the original Spikformer achieves an accuracy of 73.7%. When enhanced with LDD, the accuracy significantly improves to 78.8%. The baseline Spike-driven Transformer achieves a top-1 accuracy of 74.6%. With the LDD enhancement, the accuracy increases to 79.4%. The original Meta-SpikeFormer achieves an accuracy of 77.2%. After incorporating LDD, the top-1 accuracy reaches 80.9%.

CIFAR10 [41]: The CIFAR10 dataset, introduced by Krizhevsky et al. in [41], is a collection of images frequently used for training machine learning and computer vision algorithms. It is among the most extensively utilized datasets in machine learning research. It contains a total of 10 categories of RGB color images: airplane, automobile, bird, cat, deer, dog, frog, horse, ship, and truck. Each image has a size of 32 × 32 pixels. The batch size is set to 256. A two-block SPS splits the image into 64 patches of size 4 × 4. Table 1 shows the accuracy of different models compared with baseline methods on CIFAR10. Spikformer + LDD (94.6%) outperforms the original Spikformer by 1.1%. Spike-driven Transformer + LDD (95.6%) shows a 1.2% improvement over the original Spike-driven Transformer. Meta-SpikeFormer+LDD (96.1%) achieves a 0.7% increase compared to the original Meta-SpikeFormer.

CIFAR100 [41]: The CIFAR100 dataset, introduced by Krizhevsky et al. in [41], is widely used for training and testing deep learning models. Composed of 60,000 32 × 32 color images, CIFAR100 has 100 classes divided into 20 superclasses. Each class contains 600 images. The dataset includes two labels: fine_labels and coarse_labels, representing fine-grained and coarse-grained labels of the images, respectively. Each class has 500 training images and 100 test images. The batch size is 256 and optimizer is AdamW with 300 training epochs. The scaling factor is 0.2 when training on CIFAR100. The accuracy of Spikformer+LDD (79.2%) is 1.3% higher than the original Spikformer (77.9%). The enhanced Spike-driven Transformer + LDD (81.5%) shows a notable improvement of 3.1% over the original Spike-driven Transformer (78.4%). The accuracy of Meta-SpikeFormer+LDD (82.3%) is 3.0% higher than the original Meta-SpikeFormer (79.3%).

The results in Table 1 indicate that our method can be widely applied to training large-scale SNN networks, achieving promising results across all datesets, Transformer structures, and time steps. Particularly for the challenging ImageNet dateset, where SNN training is difficult, applying the LDD method resulted in a significant improvement in accuracy, reaching state-of-the-art (SOTA) performance. The LDD method’s success is not limited to a specific type of Transformer structure. It performs well across various architectures, including Spikformer, Spike-driven Transformer and Meta-SpikeFormer, demonstrating its adaptability.

To eliminate the influence of network parameters, we employed the same network structure for different methods on each dateset, and compared layer-wise distillation with direct training. It is evident that, under the same parameters, our accuracy surpasses the corresponding baseline models significantly. For instance, using the Spiking Transformer-8-512 structure on the ImageNet dateset, LDD improved the model accuracy by 4.8%, demonstrating a substantial enhancement in performance. One of the key advantages of the LDD method is its ability to capture and utilize the temporal dynamics of SNNs. Unlike traditional methods that might normalize spike timing and lose temporal features, LDD leverages these dynamics, aligning SNN tokens with ANN class tokens and ensuring robust feature representation. This approach not only preserves the temporal characteristics of SNNs, but also enhances their alignment with ANNs, leading to better performance.

### 6.2. Ablation Experiments

We propose constructing the Dual SNN through projecting the SNN onto the time-less ANN, thereby aligning the ANN’s knowledge onto the entire time dimension of the SNN. This achieves alignment between the continuous-valued ANN features without a time dimension and the discrete SNN features. To verify the effects of the temporal layers and Dual SNN, we conducted an ablation experiment on both the CIFAR10 and CIFAR100 datesets.

As shown in Table 2, when taking the average across the temporal dimension or using the data from the last moment as the result of the temporal dimension projection, the SNN of Spiking Transformer-8-512, an eight-block Spikformer model with 512 hidden units, only achieved 96.2% on CIFAR10 and 81.6% on CIFAR100. The significant improvement in accuracy when using the Dual SNN (96.8% on CIFAR-10 and 83.3% on CIFAR-100) compared to simpler projection methods underscores the effectiveness of this approach. This demonstrates that leveraging the temporal dynamics of SNNs is crucial for achieving high performance, especially in handling more complex datasets like CIFAR100.

The ablation experiment provides clear evidence of the benefits of the temporal layers. Temporal layers play a crucial role in capturing the dynamic behavior of SNNs. By averaging across the temporal dimension or using the data from the last moment, we lose the rich temporal dynamics inherent in SNNs. This simplification leads to a significant drop in performance, as evidenced by the lower accuracy on CIFAR-10 and CIFAR-100 datasets. The Dual SNN construction effectively bridges the gap between continuous-valued ANN features and discrete SNN features. By aligning ANN knowledge with the entire time dimension of the SNN, the Dual SNN preserves the temporal information, leading to better feature representation and higher accuracy.

### 6.3. Deep SNN Experiments

Through the alignment of ANN and SNN features and the correction of gradient errors, LDD enhances the sample efficiency and feature precision in deep spike-based Transformer networks. This improvement enables more effective utilization of training samples and deeper structure. Constrained by the relatively small inductive bias of Transformers themselves, compared to the stronger reliance of residual networks on large-scale datesets, previous studies have shown that, without external data, the ImageNet dateset only supports training spike Transformer networks with no more than ten blocks, resulting in low sample efficiency.

Table 3 demonstrates that, with the inclusion of the LDD method, the number of blocks can be extended to 24 using the ImageNet dateset. The Spiking Transformer-12-512 achieves a slightly lower accuracy of 78.1%, while the Spiking Transformer-24-512 shows a more significant drop in accuracy to 75.2%. To our knowledge, we are the first to successfully train such a deep SNN Transformer network. The results in Table 3 highlight the stark contrast between traditional methods and the LDD-enhanced approach. Traditional methods, constrained by the inductive bias of Transformers, struggle to train deep networks effectively. In contrast, the LDD method overcomes these constraints, leading to substantial improvements in both sample efficiency and network depth. This breakthrough could pave the way for more energy-efficient deep learning models, as SNNs inherently consume less power than conventional ANNs.

**Figure 4:**
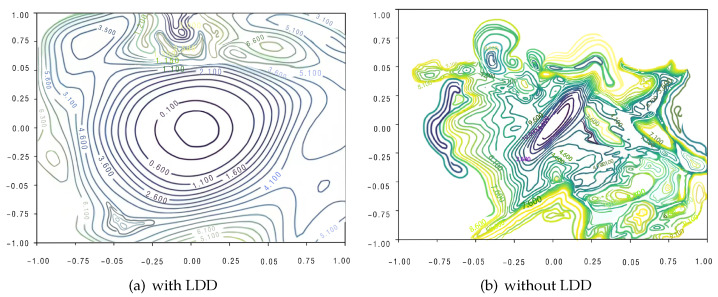
The loss surfaces of Spikformer-4-384 with/without LDD. The loss surface with LDD is smoother, indicating that the LDD method effectively reduces high-frequency oscillations in the model’s feature space, making the loss function smoother and more stable.

The LDD method also smooths the landscape of the loss function optimization, making deep SNNs converge more easily. To illustrate this, we visualize the optimization landscape of Spikformer-4-384 with and without the LDD method, as shown in Figure 4.

In Figure 4, we use the “Filter Normalization” (FN) method [42] to visualize the loss landscape of the network. We select network parameter values as the central point for visualization. By computing the values of the loss function corresponding to changes in parameters within a low-dimensional space around this central point, we can project the parameter space into a lower dimension and visualize the loss landscape in this low-dimensional space. By analyzing the geometric features of the landscape, such as sharpness or flatness, we can compare the generalization and robustness of the network.

The experiment is conducted on CIFAR-10 with Spikformer-4-384. We train the Spikformer-4-384 using AdamW optimizer. The learning rate is set as 0.001, and weight decay is set as 1e-3. We train the DNNs for 150 epochs with batch size of 128. It can be observed that the optimization landscape of the loss function is significantly smoothed with the LDD method.

## 7. Conclusions

We proposed the layer-by-layer Dual Distillation (LDD) method, which fully incorporates the supervisory information from ANNs in the training process, offering a groundbreaking solution for training deep SNN Transformers. Through designing the Dual SNN, this method aligns the output of the spiking neural networks with the features of ANNs across both spatial and temporal dimensions. Through layer-by-layer distillation, knowledge from large-scale pretrained ANNs is efficiently transferred into SNNs, effectively mitigating the issues of feature space quantization loss and inaccuracies in surrogate gradients. This approach not only ensures the low power consumption and low latency benefits of SNNs are maintained, but also enhances the depth and accuracy of these networks, improving their performance on complex datesets such as ImageNet. Our method can be broadly applied to various Transformer architectures, achieving state-of-the-art performance on standard datesets and training the deepest SNN Transformer network using the ImageNet dateset.

## Figures and Tables

**Table 1:** Comparison with baselines on CIFAR10, CIFAR100, and ImageNet.

Dataset	Method	Architecture	Time Step	Acc
CIFAR10	Spikformer [23]	Spikformer-4-384	2	93.5
	**Spikformer + LDD (Ours)**	Spikformer-4-384	2	**94.6**
	Spike-driven Transformer [22]	Spiking Transformer-4-384	2	94.4
	**Spike-driven Transformer + LDD (Ours)**	Spiking Transformer-4-384	2	**95.6**
	Meta-SpikeFormer [40]	Meta-Spikformer-4-384	2	95.4
	**Meta-SpikeFormer + LDD (Ours)**	Meta-Spikformer-4-384	2	**96.1**
CIFAR100	Spikformer	Spikformer-4-384	4	77.9
	**Spikformer + LDD (Ours)**	Spikformer-4-384	4	**79.2**
	Spike-driven Transformer	Spiking Transformer-4-384	4	78.4
	**Spike-driven Transformer + LDD (Ours)**	Spiking Transformer-4-384	4	**81.5**
	Meta-SpikeFormer	Meta-Spikformer-4-384	4	79.3
	**Meta-SpikeFormer + LDD (Ours)**	Meta-Spikformer-4-384	4	**82.3**
ImageNet	Spikformer	Spikformer-8-512	4	73.7
	**Spikformer + LDD (Ours)**	Spikformer-8-512	4	**78.8**
	Spike-driven Transformer	Spiking Transformer-8-512	4	74.6
	**Spike-driven Transformer + LDD (Ours)**	Spiking Transformer-8-512	4	**79.4**
	Meta-SpikeFormer	Meta-Spikformer-8-512	4	77.2
	**Meta-SpikeFormer + LDD (Ours)**	Meta-Spikformer-8-512	4	**80.9**

**Table 2:** Ablation studies on the temporal layer.

Architecture	Dateset	Dill	Ave	Max	Temp (Ours)
Spikformer-8-512	CIFAR10	95.6	96.1	95.4	**95.9**
	CIFAR100	80.3	79.6	80.7	**81.5**
Spiking Transformer-8-512	CIFAR10	96.4	96.5	96.2	**96.8**
	CIFAR100	80.7	81.4	81.6	**83.3**

**Table 3:** Studies on training of deep SNN Transformer.

Model	LDD	Acc
Spiking Transformer-10-512	×	74.6
Spiking Transformer-8-512	✓	78.7
Spiking Transformer-12-512	✓	78.1
Spiking Transformer-24-512	✓	75.2

## Data Availability

The original contributions presented in the study are included in the article; further inquiries can be directed to the corresponding authors.

## Abbreviations

The following abbreviations are used in this manuscript:

ANNs	Artificial Neural Networks
SNNs	Spiking Neural Networks
LDD	Layer-by-layer Dual Distillation
LIF	Leaky-Integrate-and-Fire
LSTM	Long Short-Term Memory
GRU	Gated Recurrent Unit
VSA	Vanilla Self-Attention
MLP	Multi-Layer Perceptron
SPS	Spiking Patch Splitting
SDSA	Spike-Driven Self-Attention
PSM	Patch Splitting Module
RPE	Relative Position Embedding
